# Protective Effects of Polydatin from *Polygonum cuspidatum* against Carbon Tetrachloride-Induced Liver Injury in Mice

**DOI:** 10.1371/journal.pone.0046574

**Published:** 2012-09-28

**Authors:** Hong Zhang, Cheng-Hao Yu, Yi-Ping Jiang, Cheng Peng, Kun He, Jian-Yuan Tang, Hai-Liang Xin

**Affiliations:** 1 Key Laboratory of Standardization of Chinese Herbal Medicines of Ministry of Education, Pharmacy College, Chengdu University of Traditional Chinese Medicine, Chengdu, People's Republic of China; 2 Department of Pharmacognosy, School of Pharmacy, Second Military Medical University, Shanghai, People's Republic of China; 3 Center for Drug Evaluation, State Food and Drug Administration, Beijing, People's Republic of China; 4 Department of Traditional Chinese Medicine, Changhai Hospital, Second Military Medical University, Shanghai, People's Republic of China; University of Texas Medical Branch, United States of America

## Abstract

Polydatin is one of main compounds in *Polygonum cuspidatum*, a plant with both medicinal and nutritional value. The possible hepatoprotective effects of polydatin on acute liver injury mice induced by carbon tetrachloride (CCl_4_) and the mechanisms involved were investigated. Intraperitoneal injection of CCl_4_ (50 µl/kg) resulted in a significant increase in the levels of serum aspartate aminotransferase (AST), alanine aminotransferase (ALT) and hepatic malondialdehyde (MDA), also a marked enhancement in the expression of hepatic tumor necrosis factor-alpha (TNF-α), interleukin-1 beta (IL-1β), cyclooxygenase-2 (COX-2), inducible nitric oxide synthase (iNOS) and nuclearfactor-kappa B (NF-κB). On the other hand, decreased glutathione (GSH) content and activities of glutathione transferase (GST), superoxide dismutase (SOD), catalase (CAT) and glutathione peroxidase (GPx) were observed following CCl_4_ exposure. Nevertheless, all of these phenotypes were evidently reversed by preadministration of polydatin for 5 continuous days. The mRNA and protein expression levels of hepatic growth factor-beta1 (TGF-β_1_) were enhanced further by polydatin. These results suggest that polydatin protects mice against CCl_4_-induced liver injury through antioxidant stress and antiinflammatory effects. Polydatin may be an effective hepatoprotective agent and a promising candidate for the treatment of oxidative stress- and inflammation-related diseases.

## Introduction

Hepatitis is one of the most common liver diseases and characterized by the presence of inflammatory cells in the tissue of the organ. There exist a lot of causes of hepatitis, such as immune cells in the body attacking the liver and causing autoimmune hepatitis, infections from hepatitis viruses, bacteria, or parasites, liver injury from alcohol, poisons, or hepatotoxic drugs. Hepatic inflammation is responsible for liver cells damage, fibrosis, and cirrhosis. Although there are many chemical drugs for treating these diseases, limited efficacy is obtained. Now people are becoming increasingly interested in botanical drugs for their low toxicity and good therapeutic performance. The search for new drugs capable of alleviating liver injury with fewer side effects has gained momentum over the years, creating numerous reports on significant hepatoprotective activity of plant drugs [1]–[5].

Although plant extracts constitute potential candidates, they often contain highly complex mixtures of many compounds, many of which incompletely known. Therefore more attention is focused on bioactive components from plant drugs in recent years. *Polygonum cuspidatum* sieb. et Zucc. is a medicine and food dual purpose plant, native to eastern Asia in Japan, China and Korea, whose young stems and shoots are edible as a spring vegetable, with a flavor much like mild rhubarb. The roots and rhizomes of *Polygonum cuspidatum* are safe and effective in the treatment of many diseases. Many investigations show that this plant has the properties of anti-diabetes [6], anti-hepatitis B virus [7], anti-bacteria [8], anti-inflammation [9], antioxidant [10], and so on.

Extensive chemical studies indicate that polydatin is one of the main bioactive constituents, which can inhibit the rat brain inflammatory response induced by permanent middle cerebral artery occlusion [11], protect learning and memory impairments in a rat model of vascular dementia [12], suppress UVB-induced cyclooxygenase-2 expression in vitro and in vivo via reducing the production of reactive oxygen species (ROS) [13], induce apoptosis in human nasopharyngeal carcinoma CNE cells by regulation of ROS production and mitochondrial function [14], attenuate lipopolysaccharide-induced lung injury in vivo and in vitro [15]. Polydatin exhibits favorable fall hematic fat action, which decreases the serum levels of total cholesterol (TC), triglyceride (TG) and low-density lipoprotein cholesterol (LDL-C), reduces the content of hepatic TG, and depresses the ratios of LDL-C/high-density lipoprotein cholesterol (HDL-C) and TC/HDL-C in hyperlipidemic hamster [16] and rabbit models [17]. CCl_4_-induced rat hepatocyte injury in vitro can also be evidently alleviated by polydatin via regulation of SOD and MDA levels [18]. Nevertheless, there have been no reports on the protective effects of polydatin against liver injury in vivo, as far as we are aware.

In the current study, the mouse model of CCl_4_-induced liver injury was employed, and serum transaminases, alanine aminotransferase (ALT) and aspartate aminotransferase (AST), were measured. Meanwhile, the antioxidant and antiinflammatory parameters, including hepatic SOD, MDA, GSH, GPx, GST, CAT, TNF-α, IL-1β, COX-2, iNOS, TGF-β_1_, and NF-κB, were investigated. The aims of the present study were to assess whether polydatin from *Polygonum cuspidatum* possesses in vivo protective effects against CCl_4_-induced liver injury in mice and to explore the probable mechanisms of action involved.

## Results

### Effects of polydatin on elevated serum transaminases

Acute liver injury can be indicated by elevated serum ALT and AST levels in mice 16 h after treatment with CCl_4_. As shown in Table 1, the activities of serum AST and ALT dramatically increased in the CCl4 group of mice in comparison with the control group of mice given only olive oil (43.4 and 9.1 fold, respectively; both p<0.01). However, preadministration of different doses of polydatin (25, 50, 100 mg kg^−1^ d^−1^) for 5 consecutive days remarkably reduced the elevation of serum ALT and AST levels in mice in a dose-dependent manner (ALT: 1.5, 2.4 and 3.1 fold, respectively, versus the CCl4 group; all p<0.01. AST: 1.17, 2.1 and 2.9 fold, respectively, versus the CCl4 group; p<0.01). There were no evident differences in the serum AST level between the bifendate group and the high dose group (p>0.05).

**Table 1:** Effects of polydatin on serum transaminases in CCL_4_-injured mice.

Group	Animal	Dose(mg/kg)	ALT (U/L)	AST (U/L)
Control	10	Distilled water	61.1±8.7	181.3±31.7
CCl4	10	Distilled water	2654.7±443.6ΔΔ	1648.6±265.2ΔΔ
Bifendate+CCl4	10	150	583.4±102.3**	467.5±93.6**
Polydatin+CCl4	10	25	1822.9±647.5**	1412.6±317.4
Polydatin+CCl4	10	50	1105.7±329.8**	768.7±201.5**
Polydatin+CCl4	10	100	854.4±137.1**	566.8±99.4**

Serum ALT and AST levels were markedly enhanced after 16 h of intraperitoneal injection of CCl4 (50 µl/kg), but significantly reduced by pretreatment with different doses of polydatin for 5 consecutive day.

ΔΔ p<0.01 compared with the control group;

** p<0.01 compared with the CCl4 group. Data are expressed as the mean ± SD.

### Polydatin alleviated CCl_4_-induced oxidative stress

In order to substantiate whether polydatin protects mice against CCl_4_-induced oxidative stress, the GSH level, MDA production and the activities of four antioxidative enzymes (GPx, CAT, GST, and SOD) were measured. After 16 h of CCl_4_ treatment, the production of MDA markedly increased in the CCl4 group of animals (1.6 fold) when compared with the control group of animals (p<0.01). However this increase was reversed by preadministration of different doses of polydatin, which evidently depressed the level of the hepatic MDA except in the low dose group (1.1, 1.4 and 1.5 fold, respectively, versus the CCl4 group; p<0.01). There existed no significant differences between the middle and high dose groups (50 and 100 mg/kg polydatin) and the bifendate group (p>0.05; Table 2).

**Table 2:** Effects of polydatin on hepatic biochemical parameters in CCl_4_-injured mice.

Group	Animal (N)	Dose(mg/kg)	SOD (U/mg prot)	GSH (mg/g prot)
Control	10	Distilled water	113.2±18.7	5.11±0.82
CCl4	10	Distilled water	65.6±9.7ΔΔ	3.27±0.51ΔΔ
Bifendate+CCl4	10	150	107.3±11.9**	4.78±0.79**
Polydatin+CCl4	10	25	72.9±12.3	3.49±0.47
Polydatin+CCl4	10	50	88.7±10.6**	4.44±0.61**
Polydatin+CCl4	10	100	95.4±12.1**	4.38±0.71**

GST (U/mg prot)	GPx (U/mg prot)	CAT (U/mg prot)	MDA (nmol/mg pro)
3.82±0.44	279.6±32.2	47.2±7.6	3.67±0.48
1.77±0.39ΔΔ	146.5±29.3ΔΔ	26.3±5.1ΔΔ	5.73±0.61ΔΔ
3.65±0.52**	256.4±40.7**	39.2±6.9**	4.06±0.52**
2.46±0.59*	186.4±33.9*	34.9±6.5**	5.09±0.87
2.88±0.51**	219.8±39.7**	37.7±5.7**	4.51±0.64**
3.19±0.47**	238.1±30.1**	41.3±7.1**	4.18±0.58**

Intraperitoneal injection of CCl_4_ (50 µl/kg) obviously decreased the hepatic activities of SOD, GSH, GST, GPx and CAT, and increased hepatic MDA concentration in mice. Nevertheless, the hepatic biochemical parameters involved were dramatically reversed following preadministration of different doses of polydatin for 5 consecutive days.

ΔΔ p<0.01 compared with the control group;

* p<0.05,

** p<0.01 compared with the CCl4 group. Data are expressed as the mean ± SD.

The activities of antioxidant enzymes, namely SOD, CAT, GPx and GST, in the liver are given in Table 2. A significant decrease in the activities of enzymatic antioxidants was observed in CCl_4_-treated mice (1.7, 1.8, 1.9 and 2.2 fold, respectively) in comparison with the control group of mice (all p<0.01). Pretreatment with polydatin (25, 50, 100 mg kg^−1^ d^−1^) significantly restored these functional markers toward normal in a dose-dependent manner except SOD in mice given 25 mg/kg polydatin (SOD: 1.1, 1.4 and 1.5 fold, respectively, versus the CCl4 group; p<0.01. CAT: 1.3, 1.4 and 1.6 fold, respectively, versus the CCl4 group; all p<0.01. GPx: 1.3, 1.5 and 1.6 fold, respectively, versus the CCl4 group; p<0.05 or p<0.01. GST: 1.4, 1.6 and 1.8 fold, respectively, versus the CCl4 group; p<0.05 or p<0.01). Between the middle and high dose groups (50 and 100 mg/kg polydatin) and the bifendate group, the parameters for GPx and CAT displayed unobvious differences (p>0.05).

Intraperitoneal injection of CCl_4_ also evidently reduced the level of GSH, a non-enzymatic antioxidant, in the liver of mice when compared with the control group (1.6 fold, p<0.01). But preadministration of polydatin (25, 50, 100 mg kg^−1^ d^−1^) for 5 consecutive days significantly reversed this change except in the low dose group (1.1, 1.4 and 1.3 fold, respectively, versus the CCl4 group; p<0.01). There were no statistical differences between the middle and high dose groups and the control group (p>0.05).

### Polydatin inhibited the expression of proinflammatory mediators

**Figure 1:**
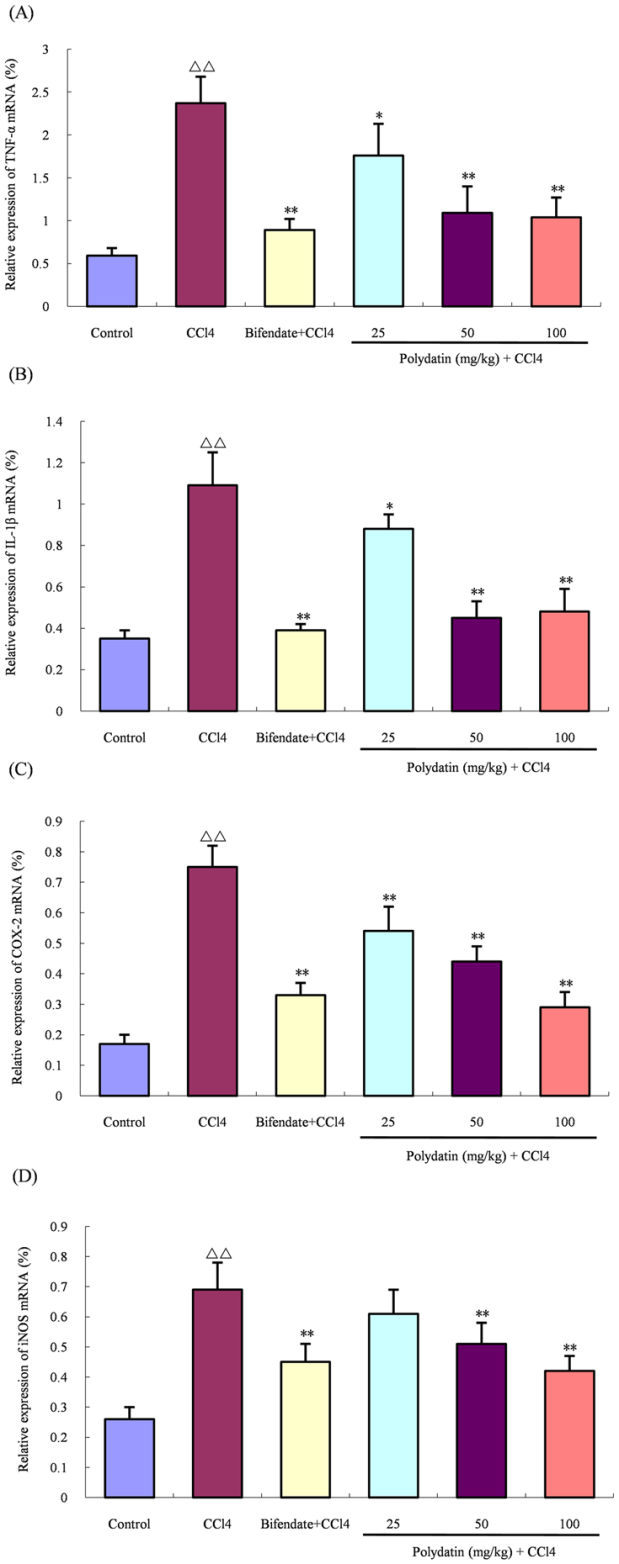
Inhibitory effects of polydatin on mRNA expression levels of proinflammatory mediators. Pretreatment with polydatin for 5 consecutive days evidently decreased mRNA expression levels of TNF-α (A), IL-β_1_ (B), COX-2 (C), and iNOS (D) prior to intraperitoneal injection of CCl_4_ (50 µg/kg). ^ΔΔ^ P<0.01 compared with the control group; *P<0.05, **P<0.01 compared with the CCl4 group. Data are expressed as the mean ± SD. n = 6.

After mice were injected intraperitoneally with 50 µl/kg of CCl_4_ for 16 h, the mRNA expression levels of proinflammatory mediators, including TNF-α, IL-1β, COX-2 and iNOS, in the liver tissue were significantly up-regulated when compared with the control group (4.0, 3.1, 4.4 and 2.7 fold, repectively; all p<0.01), suggesting induction of a severe inflammatory response. Nevertheless, different doses of polydatin markedly inhibited the mRNA expression of these proinflammatory mediators in a dose-dependent manner except iNOS in the low dose group (TNF-α: 1.4, 2.2 and 2.3 fold, respectively, versus the CCl4 group; p<0.05 or p<0.01. IL-1β: 1.2, 2.4 and 2.3 fold, respectively, versus the CCl4 group; p<0.05 or p<0.01. COX-2: 1.4, 1.7 and 2.6, respectively, versus the CCl4 group; all p<0.01. iNOS: 1.1, 1.4 and 1.6 fold, respectively, versus the CCl4 group; p<0.01) when pre-given orally to mice for 5 consecutive days (Figure 1).

### Polydatin depressed NF-κB activity

**Figure 2:**
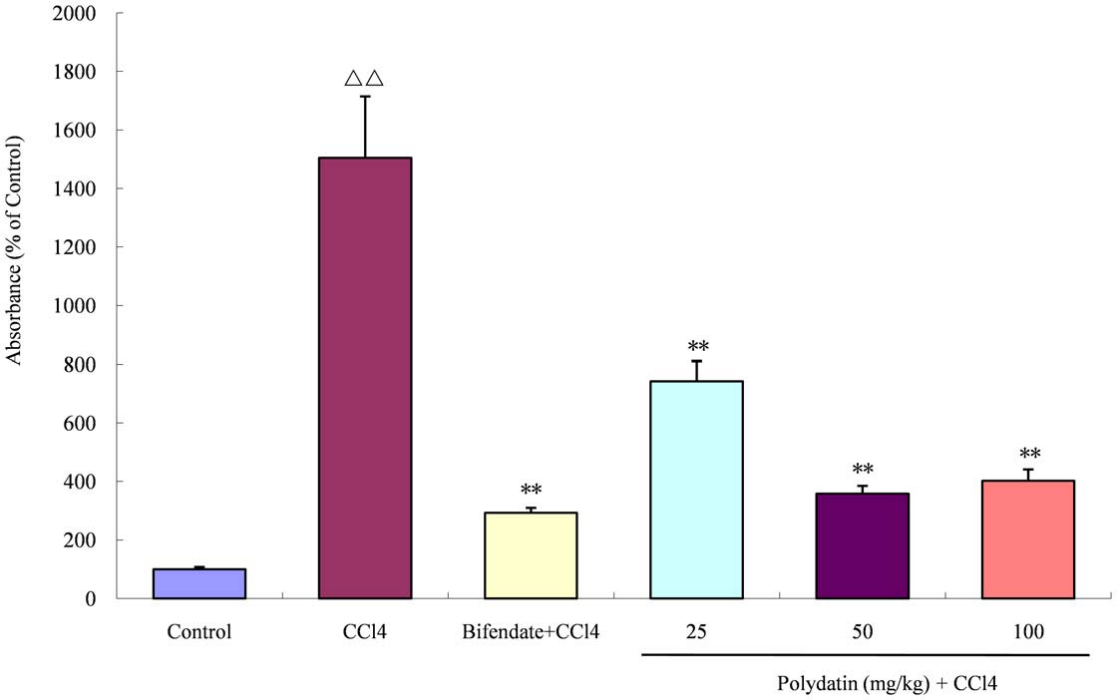
Effects of polydatin on NF-κB DNA binding activity in the liver tissue. The NF-κB activity dramatically increased in the CCl4 group of mice (50 µg/kg CCl_4_), but significantly declined following preadministration of different doses of polydatin for 5 consecutive days. ^ΔΔ^ P<0.01 compared with the control group; **P<0.01 compared with the CCl4 group. Data are expressed as the mean ± SD. n = 10.

The effects of polydatin pretreatment on the DNA binding activity of NF-κB in the liver tissue of mice are shown in Figure 2. The DNA binding activity of NF-κB was barely detected in the liver tissue of normal mice, but had a dramatic increase in the liver tissue of mice injected intraperitoneally with CCl_4_ compared with the control group of animals (15.0 fold; p<0.01). After pretreatment with different doses of polydatin for 5 days prior to intraperitoneal injection of CCl_4_, the DNA binding activity of NF-κB significantly decreased when compared with the CCl4 group (2.0, 4.2 and 3.74 fold, respectively; all p<0.01), although it was not restored to the normal level.

### Polydatin increased TGF-β_1_ expression

**Figure 3:**
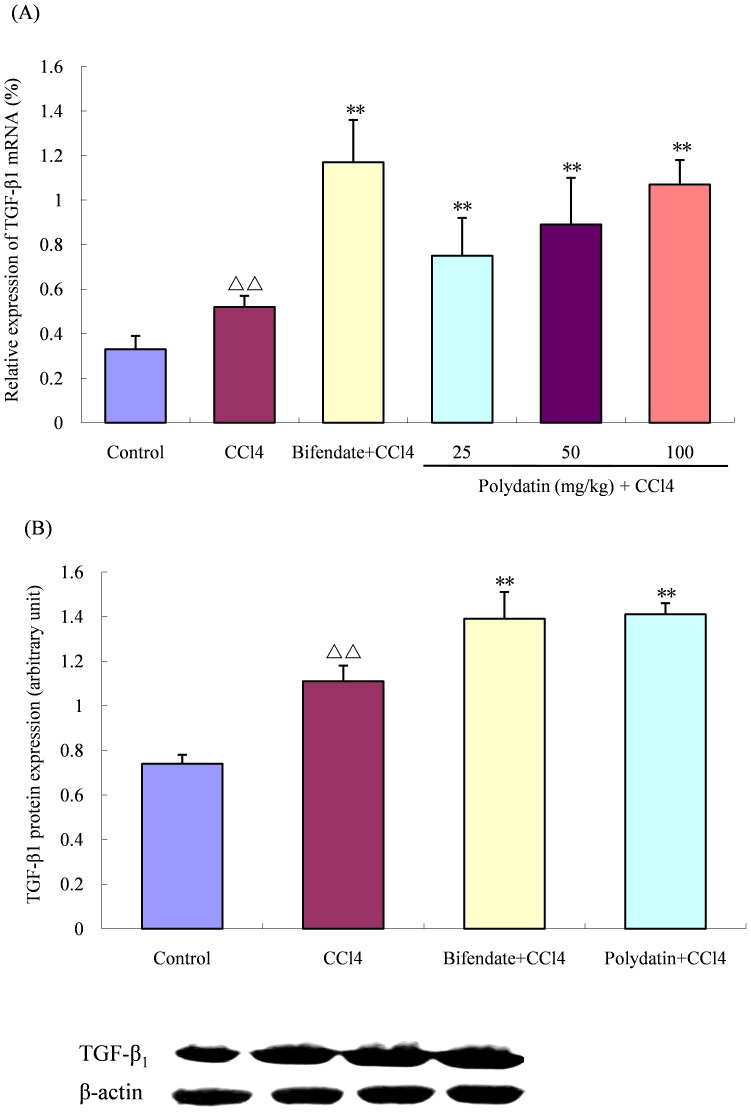
Effects of polydatin on the mRNA and protein expression of TGF-β_1_ in the liver tissue. The mRNA (A) and protein (B) expression levels of TGF-β_1_ were significantly increased in the group of mice treated with CCl_4_ (50 µg/kg) when compared with the control group. Preadministration of polydatin (100 mg/kg) for 5 consecutive days enhanced TGF-β_1_ expression levels further. ^ΔΔ^ P<0.01 compared with the control group; **P<0.01 compared with the CCl4 group. Data are expressed as the mean ± SD. 6 samples in each group were used for mRNA analysis, and 4 samples in each group for protein analysis.

Cytokine TGF-β_1_ can promote tissue repair and regeneration. In the control group of mice, TGF-β_1_ revealed lower mRNA and protein expression levels, but significantly increased in the CCl4 group of mice given 50 µl/kg CCl_4_ for 16 h (1.6 and 1.5 fold, respectively, versus the control group; both p<0.01). After pretreatment with different doses of polydatin for 5 days, TGF-β_1_ expression levels were enhanced further (mRNA expression: 1.4, 1.7 and 2.1 fold, respectively, versus the CCl4 group; all p<0.01. protein expression: 1.25 fold versus the CCl4 group; p<0.01), indicating that polydatin may promote liver tissue repair under acute injury conditions (Figure 3). There were no obvious differences in the protein expression between the polydatin group and the bifendate group (p>0.05).

### Histological findings

**Figure 4:**
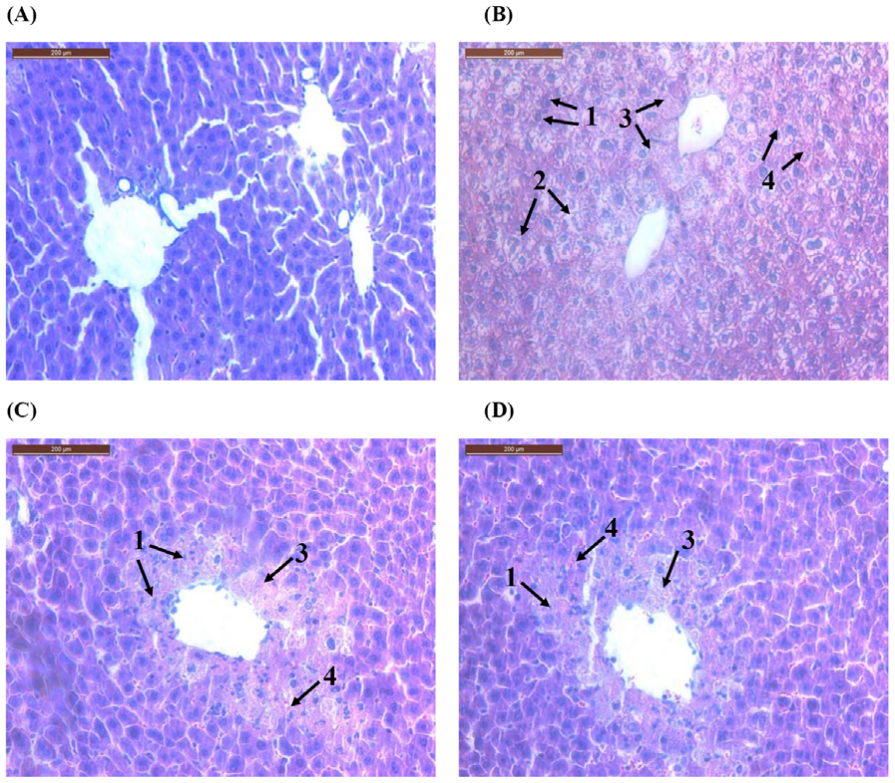
Histological characters of liver sections stained with HE. CCl4 treatment for 16 h was responsible for extensive degeneration and necrosis in the liver tissue of mice. Nevertheless, these changes were significantly attenuated by pretreatment with polydatin (D) for 5 consecutive days. (A) Control, (B) CCl4 treatment, (C) Bifendate 150 mg/kg+CCl4 treatment, (D) Polydatin 100 mg/kg+CCl4 treatment. Magnification: 200×. 1. degeneration 2. vacuole 3. necrosis 4. eosinophil infiltration.

The liver tissue showed the following histopathological changes under an optical microscope (Figure 4). The normal hepatic cells were arranged regularly and radially around central vein and the hepatic lobule structure was intact. The liver cells of mice treated with 50 µl/kg CCl_4_ appeared as degeneration, vacuoles and necrosis, and a great deal of abnormal eosinophil infiltration existed in both portal tracts and liver lobules. After administration of polydatin for 5 continuous days in advance, significantly alleviated degeneration and necrosis were observed in the liver tissue, accompanied by reduced inflammatory cell infiltration.

## Discussion

In the present study, intraperitoneal injection of CCl_4_ (50 µl/kg) markedly elicited liver injury in mice with increases in the serum levels of alanine aminotransferase (ALT) and aspartate aminotransferase (AST), enhancements in the expression levels of tumor necrosis factor-alpha (TNF-α), interleukin-1 beta (IL-1β), cyclooxygenase-2 (COX-2), inducible nitric oxide synthase (iNOS) and nuclearfactor-kappa B (NF-κB) in the hepatic tissue, the elevation of the liver malondialdehyde (MDA) content, and decreases in the expression levels of superoxide dismutase (SOD), glutathione (GSH), glutathione peroxidase (GPx), glutathione transferase (GST), and catalase (CAT) in the liver tissue. However, pretreatment with polydatin at the doses of 25, 50, and 100 mg kg^−1^ d^−1^ for 5 consecutive days prior to CCl_4_ injection reversed these changes. The mRNA and protein expression levels of growth factor-beta1 (TGF-β_1_) in the liver tissue were also increased further by polydatin. With regard to histological examination, polydatin significantly alleviated degeneration and necrosis of hepatocytes, accompanied by decreased inflammatory cell infiltration.

CCl_4_, a potent hepatotoxicity agent, has been extensively used in the establishment of animal models of liver injury for screening the hepatoprotective activity of drugs [19]–[21]. ALT and AST are reliable markers for liver function [22]. It has been established that AST can be found in cardiac muscle, skeletal muscle, kidney, brain, pancreas, lung, leukocytes, erythrocytes, especially liver, whereas ALT is present in liver tissue [23]. The dramatically elevated serum levels of transaminases AST and ALT have been attributed to damaged structural integrity of the liver. They are released into circulation when cell membrane permeability increases after the damage of hepatocytes [24]–[26]. Consistent with previous reports [2], [27], CCl_4_ markedly caused liver injury in mice with increases in the serum levels of ALT and AST in the present study. Nevertheless, pretreatment with polydatin for 5 consecutive days reversed these changes, suggesting that polydatin protected mice against CCl_4_-induced hepatotoxicity. In parallel with the alteration of liver function markers, these phenomena were also confirmed by histological findings with alleviated degeneration and necrosis and decreased inflammatory cell infiltration in the liver tissue.

CCl_4_ can elicit severe hepatocellular damage for its highly toxic metabolite trichloromethyl free radical by the action of the cytochrome P540 system. In the presence of oxygen, it is converted into a more reactive radical and initiates the process of lipid peroxidation [28]. Three enzymes, namely SOD, CAT and GPx, comprise the major antioxidant system in mammalian cells, which constitute a mutually supportive team for defense against reactive oxygen species (ROS) [29]. SOD can convert superoxide anions to H_2_O_2_, which is changed further to H_2_O with the help of GPx and CAT. SOD also inhibits hydroxyl radical production [30]. It is crucial to maintain the balance between ROS and antioxidant enzymes, which serves as a major mechanism in preventing damage elicited by oxidative stress [31]. This imbalance can produce toxic effects within the body and damage all the single aspects of a cell, including its protein, lipids and DNA.

Non-enzymatic antioxidants, such as GSH, also play an excellent role in protecting cells from CCl_4_-induced hepatotoxicity. GSH can be combined with the toxic metabolite of CCl_4_, namely trichloromethyl radical, in the presence of GST catalytic activity, which contributes to detoxification of CCl_4_. GSH stores are markedly depleted, especially when liver necrosis initiates [26], [32]. Therefore, one of the principal causes of CCl_4_-induced liver injury is lipid peroxidation caused by its free radical derivatives [26].

In the liver tissue of CCl_4_-treated rats, lipid peroxidation significantly increases and the activity of antioxidant enzymes is inhibited [33], [34], which is also substantiated by our experiment results in mice, but polydatin significantly enhanced liver tissue mRNA expression levels of SOD, GSH, GPx, GST and CAT in liver injury mice induced by CCl_4_, indicating its favorable antioxidant activity. These results are consistent with previously reported antioxidant effects of polydatin. The research by Li et al. [12] displays that oral administration of polydatin for 30 days can protects vascular dementia rats from learning and memory impairments through a decrease in MDA production and increases in SOD and CAT activities. Elevation of oxidative agents and decline in anti-oxidant substance can promote ischemia/reperfusion injury, but polydatin exerts cardioprotective effects against this damage via enhancement of SOD activity and reduction of MDA content [35]. Regulation of SOD and MDA levels by polydatin also significantly alleviates CCl_4_-induced rat hepatocyte injury in vitro [18]. These investigations suggest the direct anti-oxidative stress mechanisms of polydatin.

On the other hand, CCl_4_ can also rapidly promote the expression of proinflammatory mediators and chemokines from hepatic Kupffer cells to exert paracrine actions [36], [37], which is responsible for the recruitment of circulating immune cells as part of the inflammatory response, but the antioxidant system is required for attenuation of the inflammatory response in liver, suggesting there exists a link between oxidative stress and inflammation [38]. In the current study, we found that the mRNA expression levels of proinflammatory mediators, including TNF-α, IL-1β, iNOS and COX-2, evidently increased in the liver tissue of mice treated with CCl_4_ for 16 h, but preadministration of polydatin for 5 days markedly decreased their expression. These results suggest that polydatin not only inhibits the hepatic local inflammatory response, but also attenuates the positive feedback loop between oxidative stress and inflammation.

Most recently, Ji et al. [11] reported that polydatin inhibited the brain local inflammatory response in brain damage rats by decreasing NF-κB activation and oxidative stress, which is in agreement with our current findings. Accumulation of the NF-κB p65 subunit in the liver cells of CCl_4_-treated mice was significantly decreased by preadministration of polydatin for 5 consecutive days. Cytoplasmic NF-κB activation and its subsequent nuclear translocation induced by ROS are responsible for aggravation of liver injury by influencing cytotoxic cytokine production, such as TNF-α [39], followed by activation of other inflammatory mediators, such as COX-2 and iNOS [40], [41]. Thus NF-κB plays a central role in the process of inflammation, and the antiinflammatory activity of polydatin in liver injury mice was achieved probably through modulation of NF-κB.

In the process of liver regeneration, TGF-β_1_ is important for the assembly of hepatic tissue [42], [43], which promotes the growth of hepatic stellate cells and hepatocytes [42]. In our study, similar to the previous report [37], intraperitoneal injection of CCl_4_ was responsible for increased mRNA expression of TGF-β_1_ in the hepatic tissue of mice, which was enhanced further by pradministration of polydatin, suggesting its facilitating effects on liver regeneration after acute liver injury.

In conclusion, our investigation demonstrates, for the first time, in vivo hepatoprotective activity of polydatin from *Polygonum cuspidatum*, which attenuates hepatic oxidative stress and inhibits inflammation in CCl_4_-injured liver tissue of mice. These effects are achieved through decreased levels of MDA, TNF-α, IL-1β, COX-2, iNOS and NF-κB, and enhanced levels of SOD, GSH, GPx, GST, CAT and TGF-β_1_ in the liver tissue. Our findings suggest that consumption of *Polygonum cuspidatum* and relative products should therefore be encouraged not only in healthy people, but especially in those with increased risk of toxic liver damage.

## Materials and Methods

### Ethics statement

All animal treatments were strictly in accordance with international ethical guidelines and the National Institutes of Health Guide concerning the Care and Use of Laboratory Animals, and the experiments were carried out with the approval of the Committee of Experimental Animal Administration of the Second Military Medical University (approval number: 1200376).

### Preparation of polydatin

The root and rhizome of *Polygonum cuspidatum*, obtained from Shanghai Tong-ren-tang Pharmaceutical Group, were identified by Professor H.-C. Zheng, a pharmacognosist, from the School of Pharmacy of the Second Military Medical University (Shanghai, China). A voucher specimen was deposited with the number SY3629 in the Department of Pharmacognosy, School of Pharmacy, Second Military Medical University.

High-speed counter-current chromatography (HSCCC) was applied to separation and purification of polydatin from *Polygonum cuspidatum* as described by Du et al [16]. Briefly, dry mass of the plant was extracted by methanol, the mixture was centrifuged and the supernatant was washed with light petroleum (b.p. 60–90°C). The remaining methanol phase was evaporated to form syrup. The syrup was then dissolved and fractionated in EtOAc and water in the ratio of 1∶1. Then water solution was vacuum evaporated at 40°C and the crude extract was obtained.

The proper amount of the crude extract was injected into HSCCC, and after two separation procedure with EtOAc–EtOH–water (10∶1∶10 and 70∶1∶70, v/v), respectively, polydatin was obtained from *Polygonum cuspidatum* with the yield of 0.98% (Molecular formula C_20_H_22_O_8_; molecular weight: 390.40; purity: 93.7%).

### Animals and treatments

Sixty male ICR mice, purchased from Shanghai Si-Lai-Ke Experimental Animal Ltd. (Shanghai, China) with an initial body weight of 19–21 g, were used in this study. They were housed in a regulated environment (20±1°C), with a 12-h light/dark cycle (lights on: 08:00–20:00 h). Unless specified otherwise, food and water were given ad libitum throughout the experiment.

After 3-d acclimation, animals were randomly divided into 6 groups with 10 mice in each group, that is, one control group given distilled water, one CCl4 group administered distilled water, three groups treated with polydatin and CCl4, and one positive group given bifendate (Xin Chang Pharmaceutical Factory, Zhejiang Pharmaceutical Co., Ltd.) and CCl4.

Polydatin was dissolved in distilled water prior to administration. Three treatment groups of animals were orally administered 25, 50, 100 mg kg^−1^ d^−1^ of polydatin by intubation, respectively, for 5 consecutive days. Bifendate was suspended in distilled water; animals in the positive control group were orally administered 150 mg kg^−1^ d^−1^ of bifendate for 5 consecutive days in the same way. Other groups of animals were orally administered distilled water of same volume, and they were run concurrently with polydatin-treated groups.

After 1 h of the last administration, all animals were given intraperitoneally 50 µl/kg of CCl_4_ dissolved in olive oil except mice in the control group. 16 hours later, animals were decapitated and blood was collected for the test of serum ALT and AST levels. The livers were immediately removed and cleaned in 0.9% sodium chloride (4°C), and a portion was stored at −80°C for further RNA extraction.

### Measurement of serum ALT and AST

Serum samples were separated from blood by centrifugation at 1500 g for 15 min, and aspartate aminotransferase (AST) and alanine aminotransferase (ALT) were measured using an automated biochemistry analyzer (HITACHI 7600-020, Tokyo, Japan) to assess the hepatic function.

### Hepatic biochemical assays

Liver tissue was homogenized with corresponding buffer according to the protocols of commercially available kits (Jiancheng Institute of Biotechnology, Nanjing, China) and centrifuged at 1500 g for 20 min at 4°C. The supernatant was used for the measurement of glutathione transferase (GST), superoxide dismutase (SOD), catalase (CAT), glutathione peroxidase (GPx), glutathione (GSH) and malondialdehyde (MDA) following the commercial kit instructions.

### Fluorescent quantitative reverse transcription-PCR (FQ-RT-PCR)

TriPure Isolation Reagent (Roche Diagnostics, Vilvoorde, Belgium) was used to extract the total mRNA of the liver tissue in accordance with our previous report [44], and the isolated RNA was treated with RNase-free DNase (Promega). Reverse transcription was performed using a cDNA synthesis kit according to the manufacturer's instructions (Applied Biosystem).

Primer pairs for mice genes including tumor necrosis factor-alpha (TNF-α), interleukin-1 beta (IL-1β), cyclooxygenase-2 (COX-2), inducible nitric oxide synthase (iNOS), growth factor-beta1 (TGF-β_1_) and nuclearfactor-kappa B (NF-κB) were designed using the Primer Express design software (Applied Biosystems) and listed in Table 3. The house-keeping gene GAPDH was used as an internal control. FQ-RT-PCR was performed on a real-time PCR instrument (ABI 7900HT, Applied Biosystems) for 40 cycles consisting of denaturation at 95°C for 30 s, annealing at 59°C for 30 s and extension at 72°C for 30 s. All amplifications and detections were carried out in a MicroAmp optical 96-well reaction plate with optical adhesive covers (Applied Biosystems). Relative expression of mRNA (%) = 2^−ΔCT(1,2,3,4,5)^×100%, where CT represents threshold cycle, ΔCT_1_ = CT_(TNF-α)_−CT_(GAPDH)_, ΔCT_2_ = CT_(TGF-β1)_−CT_(GAPDH)_, ΔCT_3_ = CT_(IL-1β)_−CT_(GAPDH)_, ΔCT_4_ = CT_(COX-2)_−CT_(GAPDH)_, ΔCT_5_ = CT_(iNOS)_−CT_(GAPDH)_.

**Table 3:** Primers used in real-time RT-PCR analysis.

Gene	Primer sequence	Species	Amplicon size (bp)
TNF-α	Forward: 5′ GGCAGGTCTACTTTGGAGTC 3′	Mouse	233
	Reverse: 5′ CACTGTCCCAGCATCTTGTG 3′		
IL-1β	Forward: 5′ GCAGGCAGTATCACTCATTG 3′	Mouse	165
	Reverse: 5′ CACACCAGCAGGTTATCATC 3′		
iNOS	Forward: 5′ GCCCTGCTTTGTGCGAAGTG 3′	Mouse	144
	Reverse: 5′ AGCCCTTTGTGCTGGGAGTC 3′		
COX-2	Forward: 5′ CCTGGTCTGATGATGTATGC 3′	Mouse	121
	Reverse: 5′ GTATGAGTCTGCTGGTTTGG 3′		
TGF-β_1_	Forward: 5′ AAGGACCTGGGTTGGAAGTG 3′	Mouse	125
	Reverse: 5′ TGGTTGTAGAGGGCAAGGAC 3′		
GAPDH	Forward: 5′ CATCAACGGGAAGCCCATC 3′	Mouse	211
	Reverse: 5′ CTCGTGGTTCACACCCATC 3′		

### Assessment of NF-κB DNA binding activity

NF-κB DNA binding in nuclear extracts was determined using a commercially available NF-kB p65 ELISA kit following the manufacturer's instructions (TransAM ELISA kit, Active Motif) and a previous report [45]. Nuclear protein was obtained from tissue lysates prepared from fresh liver tissue of mice. The amount of bound NF-kB p65 was detected by adding IRDye conjugated secondary antibody. Optical density value was read at 450 nm using colorimetric detection in a 96 well ELISA plate reader. All results are expressed as averages of the two duplicate samples assayed after subtracting the average of the two blank values.

### Western blot assay

The liver tissue was homogenized in lysis buffer and centrifuged. Supernatants were used to estimate protein concentrations.TGF-β_1_ protein expression was detected by Western blot hybridization according to the manufacturer's instructions and previous reports [46]–[48].

### Histopathological examination

Liver samples were obtained from animals, fixed with 10% buffered formalin for 3 days. Tissue pieces were washed in running water, dehydrated in alcohol, embedded in paraffin, and sectioned with a dermatome. 5 µm thick tissue sections were deparaffinized with dimethylbenzene for 10 min, rehydrated for 10 min through gradient ethanol immersion, and washed in running water for 3 min. The tissue was stained in hematoxylin working solution for 10 min before rinsing the sections for 2 minutes in running water. Subsequently, the sections were orderly immersed in differentiation solution for 15 sec, washed for 45 min in running water, stained in eosine working solution for 5 min, and washed for 2 min in running water. The sections were dehydrated further for 10 min via gradient ethanol immersion, and clarified with dimethylbenzene for 10 min. Light microscopy was used to examine the mounted tissue.

### Statistical analysis

The data were analyzed using a SPSS 13.0 statistical package. Multiple comparisons were performed by one-way analysis of variance (ANOVA) followed by LSD t-test. A value of p<0.05 was considered statistically significant, and all results are presented as the mean ± SD.
